# Peripheral Neuropathy and Tear Film Dysfunction in Type 1 Diabetes Mellitus

**DOI:** 10.1155/2014/848659

**Published:** 2014-08-07

**Authors:** Stuti L. Misra, Dipika V. Patel, Charles N. J. McGhee, Monika Pradhan, Dean Kilfoyle, Geoffrey D. Braatvedt, Jennifer P. Craig

**Affiliations:** ^1^Department of Ophthalmology, New Zealand National Eye Centre, Faculty of Medical and Health Sciences, University of Auckland, Private Bag 92019, Auckland 1142, New Zealand; ^2^Department of Neurology, Faculty of Medical and Health Sciences, University of Auckland, Private Bag 92019, Auckland 1142, New Zealand; ^3^Department of Medicine, Faculty of Medical and Health Sciences, University of Auckland, Private Bag 92019, Auckland 1142, New Zealand

## Abstract

*Purpose.* To compare tear film metrics in patients with type 1 diabetes mellitus (DM) and healthy controls and investigate the association between peripheral neuropathy and ocular surface quality. *Methods.* Dry eye symptoms were quantified in 53 patients with type 1 DM and 40 age-matched controls. Ocular examination included tear film lipid layer thickness grading, tear film stability and quantity measurement, and retinal photography. DM individuals additionally underwent a detailed neuropathy assessment. *Results.* Neither mean age nor dry eye symptom scores differed significantly between the DM and control groups (*P* = 0.12 and *P* = 0.33, resp.). Tear lipid thickness (*P* = 0.02), stability (*P* < 0.0001), and quantity (*P* = 0.01) were significantly lower in the DM group. Corneal sensitivity was also reduced in the DM group (*P* < 0.001) and tear film stability was inversely associated with total neuropathy score (*r* = −0.29, *P* = 0.03). *Conclusion.* The DM group exhibited significantly reduced tear film stability, secretion, and lipid layer quality relative to the age-matched control group. The negative correlation between tear film parameters and total neuropathy score suggests that ocular surface abnormalities occur in parallel with diabetic peripheral neuropathy.

## 1. Introduction

While cataract and retinopathy have been extensively researched in patients with diabetes mellitus (DM), only a fraction of the published research has been dedicated to ocular surface complications. However, dry eye symptoms and signs of epithelial fragility, punctate keratopathy, persistent epithelial defects, and decreased corneal sensitivity are not uncommon in DM [1–3]. Compromised innervation of the cornea in patients with DM has also been described [4–6]. Tear film dysfunction, characterised by impairment in tear quantity and quality, can occur in association with abnormal corneal innervation due to the intimate, functional relationship between the cornea and the preocular tear film [7, 8]. The resulting dry eye is a recognised cause of debilitating, chronic ocular irritation symptoms [9]. The restrictions on life imposed by this chronic condition can be significant and, in terms of impact on quality of life, have been equated in more severe cases to those induced by dialysis and severe angina [10].

Tear film irregularity has been reported in DM, especially in association with that of extended disease duration and severity, as defined by stage of retinopathy [2, 11]. It has been postulated that damage to the microvasculature and denervation of the lacrimal gland may contribute to impaired lacrimation in DM [2, 7, 12]. Despite this neural link, few studies have explored whether the ocular surface alters in association with peripheral neuropathy [7, 13, 14], although anomalous innervation of the lacrimal gland has been reported in those with diabetic sensory neuropathy [2, 7].

This prospective study sought to compare the tear film in DM with that of control subjects and examine the relationship of the preocular tear film with markers of peripheral neuropathy.

## 2. Materials and Methods

The study adhered to the tenets of the Declaration of Helsinki and was conducted with Regional Ethics Committee approval (NTX/09/12/122). Two hundred and seven patients, identified by a specialist endocrinologist (Author, Geoffrey D. Braatvedt) as having a history of type 1 DM, were invited to participate. A history of laser therapy for retinopathy, cumulative contact lens wear of ≥3 months, ocular surgery, ocular trauma, or ocular or systemic disease that may affect the ocular surface, or a diagnosis of peripheral neuropathy unrelated to DM precluded participation in the study. Consequently, 53 individuals with DM were deemed eligible and were willing to participate in the study. Forty healthy, nondiabetic volunteers (HbA_1c_ < 41 mmol/mol) were recruited as controls.

**Figure 1:**
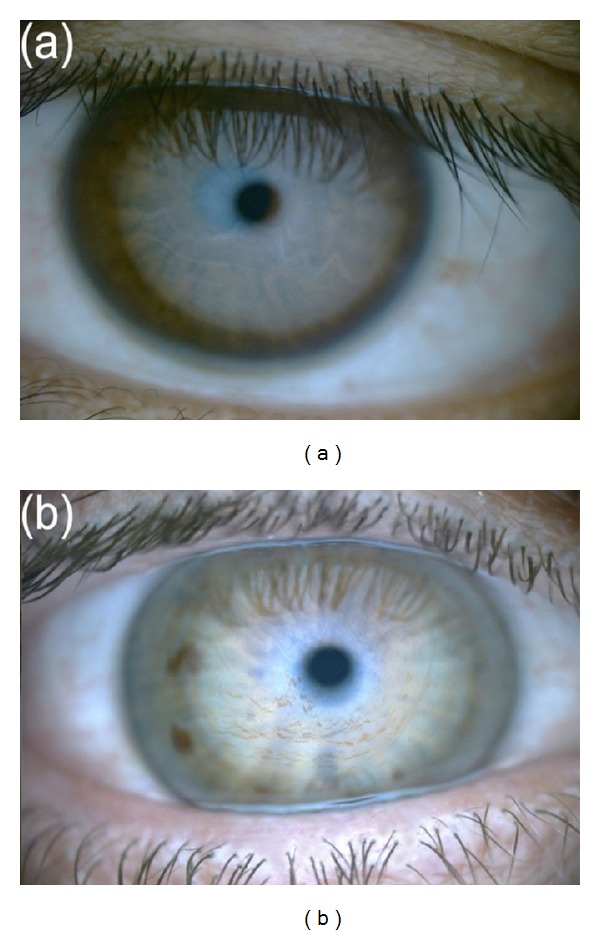
Representative examples of tear film—lipid layer grades: (a) grade 3: wave pattern; (b) grade 5: colour fringe pattern.

For each participant, a detailed ocular and general medical history, including smoking and alcohol use, was obtained. Dry eye symptoms were recorded and scored with the McMonnies dry eye questionnaire [15] and general ocular surface and eyelid examination was performed by slit-lamp biomicroscopy (Topcon Medical Systems, NJ, USA). Tear film interferometry with the Keeler Tearscope Plus (Keeler Ltd, Berkshire, UK) enabled tear film lipid patterns to be graded according to Guillon's classification [16]. Lipid patterns corresponding to increasing lipid layer thickness were subsequently assigned numerical lipid layer grades 0–5, for the purpose of statistical analysis, as 0 (absent), 1 (open meshwork), 2 (closed meshwork), 3 (wave), 4 (amorphous), or 5 (coloured fringes) (Figure 1). Tear film stability was recorded with the aid of the Tearscope Plus with the fine grid insert, as the noninvasive tear breakup time (NIBUT). An average of 3 readings was calculated for each measurement. The Phenol Red Thread (PRT) test (Zone-Quick, FCI Ophthalmics, Pembroke, MA, USA) provided an index of tear quantity. Central corneal sensitivity was measured using noncontact corneal aesthesiometry with the NCCA (Glasgow Caledonian University, UK) and adopting a double staircase method of threshold determination [17]. All ocular assessments were performed at a single visit, on the right eye only, with the exception of subjects reporting unilateral surgery or trauma to the right eye, in which case the left eye was examined. Tests were performed in the same order for each participant, from least to most invasive, to minimize the effect of reflex tearing.

Digital images of the central and peripheral retina were captured (Non-Mydriatic Retinal Camera DR-DGi, Canon Inc., USA) and graded according to the Early Treatment Diabetic Retinopathy Study (ETDRS) criteria [18] for diabetic retinopathy by an independent, fellowship-trained, medical retina specialist (Author, Monika Pradhan).

The DM group underwent neuropathic assessment by an experienced neurologist (Author, Dean Kilfoyle). An overall neuropathy score (total neuropathy score or TNS) was obtained from a previously validated and recognised combination of the symptomatic neuropathy score [19], a clinical neuropathic assessment by the neurologist, biothesiometry (quantitative sensory testing) on the medial malleolus and great toe, and nerve conduction study (NCS), with increasing score representing increasing severity of neuropathy [19].

Statistical analysis was undertaken with IBM SPSS v19.0 (Chicago, IL, USA). Regression analysis was performed between the results of the tear film assessment techniques, corneal sensitivity, diabetic retinopathy grading, and total neuropathy score. Positively skewed raw NIBUT data were logarithmically transformed prior to parametric statistical testing. One-factor ANOVA and Friedman tests were performed between variables with normally distributed and non-normally distributed data, respectively, to test differences between controls and DM groups. Pearson and Spearman correlation (2-tailed) analyses were performed for data that did and did not approximate normal distributions, respectively. A *P* value of less than 0.05 was considered statistically significant.

## 3. Results and Discussion

### 3.1. Results

Patient characteristics including gender, age, ethnicity, and duration of diabetes are described in Table 1. All but two participants in the DM group self-identified as being of European descent while the control participants included 47.5% NZ European, 22.5% Indian, and 20% Asian (excluding Indian). The mean age of the DM group (49 ± 12 years) did not differ significantly from that of the control group (44 ± 15 years) (*P* = 0.12) and no significant differences in tear film characteristics were identified between the ethnic subgroups (*P* > 0.05).

Dry eye symptom scores and clinical findings were compared for the diabetes and control groups (Table 2). The mean dry eye symptom score was not statistically significantly different between the diabetes and control groups (*P* = 0.33). However, clinical tests showed significant differences between the two groups, with lipid layer grading (*P* = 0.02), NIBUT (*P* < 0.0001), and PRT test (*P* = 0.01) results all statistically, and clinically, significantly lower in the DM group. NIBUT was observed to be lower in females than in males in both the control (*P* = 0.06) and DM (*P* < 0.01) groups. The patients with DM exhibited reduced corneal sensitivity compared to controls (*P* < 0.0001) (corneal sensitivity and subbasal nerve density reported in detail elsewhere) [20]. The total neuropathy score (maximum possible score 40) ranged between 0 and 21 (5.3 ± 5.1) in the patients with diabetes.

Retinopathy was not observed in the majority of patients (60%) while 21% exhibited mild and 19% exhibited moderate DR. Regression analysis between interferometry, tear film stability, tear quantity, diabetic retinopathy grade, and total neuropathy score (Table 3) highlighted a positive relationship between lipid layer grading and both NIBUT (*r* = 0.56, *P* < 0.01) and tear quantity (*r* = 0.38, *P* < 0.01). Tear film stability was noted to be inversely related to total neuropathy score (*r* = −0.29, *P* = 0.03). The association between corneal sensitivity and total neuropathy score failed to reach statistical significance (*r* = 0.24, *P* = 0.08).

### 3.2. Discussion

Comparison of the status of the tear film between patients with type 1 DM and age-matched healthy control subjects demonstrated a significantly poorer tear film quality in diabetes. Lipid layer grade, tear film stability, and tear quantity (basal with minimal reflex tear secretion) [21] were all significantly reduced in patients with type 1 DM, confirming compromised protection of the ocular surface in patients with diabetes.

Dry eye symptoms were observed to increase in severity with advancing age in both groups, in the current study, consistent with reports in the literature [22, 23]. Interestingly, age-matched subjects in both groups reported similar dry eye symptom severity, despite clear differences in tear quality (*P* < 0.0001). This lack of difference in symptoms is believed to be related to the impaired sensitivity of the ocular surface in diabetes.

Tear film stability and lipid layer thickness have been shown to be influenced by gender [24], with older women tending to exhibit thinner and more contaminated lipid layers [25]. The current study also reported reduced tear film stability in females compared to males, both in the DM group (*P* < 0.01) and in the control group (*P* = 0.06). A decrease in circulating androgens in postmenopausal women is believed to play an important role in affecting meibomian gland function, supporting female gender as a risk factor for dry eye disease [26].

In addition to a reduction in tear film stability, several studies have reported a diminished tear secretion in the diabetic eye [7, 11, 27], as observed in the present research. This further degrades the quality of tear film in an already compromised diabetic eye.

Reduced tear film stability has been associated with the presence of superficial punctate keratitis [8]. Functional abnormalities of corneal innervation may contribute to the incidence of superficial punctate keratitis in these patients through its adverse effect on tear film instability [28]. An inverse association between corneal innervation and peripheral neuropathy has been reported previously [6, 29]. The current study supports this relationship, with a modest inverse correlation observed between NIBUT and total neuropathy score (a measure of peripheral neuropathy) (*r* = −0.29, *P* = 0.03).

Retinopathy grade was observed to be unrelated to tear film stability in the current study (*r* = −0.03, *P* = 0.82), contrary to previous observations [11, 30]. However, it should be noted that, as history of laser treated retinopathy was an exclusion criterion in the current study, patients with more than moderate retinopathy were generally ineligible to participate. This restriction in the range of disease severity in the cohort enrolled in the current study likely contributed to the absence of a relationship in our study.

In this New Zealand-based study, the control group comprised patients of a variety of ethnic backgrounds reflecting the multiethnic population [31]. Type 1 DM in New Zealand is predominantly reported in those with European (Caucasian) heritage [32] and this was observed in the present cohort. Although ethnicity has previously been shown to be a determinant of tear film stability [33], no significant difference was observed between the ethnic subgroups of the control subjects in the present study.

There is potential for lipid, aqueous, and mucins, the major components of the tear film, to be adversely affected in patients with diabetes. The meibomian and lacrimal glands are responsible for the secretion of the lipid and aqueous portions of the preocular tear film, respectively. Meibomian glands are innervated by parasympathetic fibres with a smaller contribution from sympathetic and sensory neurons [34]. Disease or any damage to these neurons leads to dry eye in an animal model [35]. Two human studies have previously reported a compromised tear lipid layer in DM patients, as confirmed in the current study [8, 36]. Clinical observation of noncontiguous or absent lipid layers is associated with significantly increased tear film evaporation [37], one of the key factors in dry eye development [38].

Elevated expression of advanced glycation end products in the lacrimal gland has been postulated as a reason for changes in lacrimal gland function described in diabetes [12]. A reduction in goblet cell numbers, compromising mucin quantity, may also contribute to the tear film instability observed in diabetes [7, 39]. Goblet cell loss in those with diabetic peripheral neuropathy and poor metabolic control has been previously reported [7].

The reduced tear production identified in patients with diabetes (*P* = 0.01) in the current study lends support to the concept that lacrimal gland function might be adversely affected by a neuropathic mechanism [40], resulting in dysfunction of the ocular surface secretory glands via their innervation. Such dysfunction could arise from a peripheral neuropathy involving the afferent sensory nerves from the ocular surface affecting corneal sensitivity and the autonomic (efferent) nerves responsible for innervating the tear-component secreting glands and the lacrimal and meibomian glands [35, 40, 41]. This is supported by the current results of reduced corneal sensitivity; however the previously reported association between corneal sensitivity and peripheral neuropathy failed to reach statistical significance in the current study (*r* = 0.24, *P* = 0.08) [42]. Reduced tear secretion has previously been identified in patients with type 2 diabetes relative to healthy controls [8, 11].

## 4. Conclusion

In summary, the current study confirms the underlying threat to ocular surface health in patients with type 1 DM compared to control subjects. The reduction in tear production in patients with DM and the association between reduced tear film stability and diabetic peripheral polyneuropathy add credence to the hypothesis that diabetic peripheral neuropathy is associated with, or directly affects, secretory lacrimal gland function [43]. Hence, patients with DM, particularly the older female, are at a greater risk of dry eye and compromised preocular tear film.

## Figures and Tables

**Table 1:** Participant characteristics for the patients with type 1 diabetes and control group.

	Diabetes	Controls
Subjects (*n*)	53	40
M : F ratio	26 : 27	17 : 23
Age (years)	48.6 ± 11.8	44.3 ± 14.7
HbA_1c_ (mmol/mol)	61.3 ± 12.0	35.0 ± 2.5
Ethnicity		
European	51	19
Indian	0	9
Asian (excluding Indian)	1	8
Maori	1	0
Others	0	4
Mean diabetes Duration (years)	25.8 ± 11.4	
<10	5	
10–20	10	
21–31	22	
>31	16	

**Table 2:** Comparison of symptoms and tear characteristics for control and patient groups (mean/median), together with the significance of their differences (ANOVA/Friedmann). Noninvasive tear breakup time (NIBUT) values are extrapolated from logarithmically transformed data.

	Diabetes	Healthy controls	ANOVA/Friedmann (*P* values)
McMonnies questionnaire (mean ± SD)	8.8 ± 6.7	7.6 ± 4.6	0.33
Lipid layer thickness grade (median)	2	3	0.02
NIBUT (s) (mean ± SD)	6.0 ± 1.9	8.2 ± 2.5	<0.0001
Phenol red thread test (mm) (mean ± SD)	13.7 ± 4.7	16.3 ± 4.9	0.01
Corneal sensitivity threshold (mBAR)	1.3 ± 1.3	0.2 ± 1.3	<0.001

**Table 3:** Correlation analysis between age, diabetes duration, McMonnies questionnaire scores (DEQ), phenol red thread test (PRTT) (mm), tear film interferometry including lipid layer grade, stability (NIBUT), diabetic retinopathy grading, and total neuropathy score (TNS) in those with DM (*n* = 53).

	Correlation (*r* values)	Probability (*P* values)
Age versus NIBUT	−0.28∗	0.05
Age versus TNS	0.41∗∗	0.00
DEQ versus TNS	−0.05	0.73
Lipid layer thickness versus NIBUT	0.56∗∗	0.00
Lipid layer thickness versus PRTT	0.39∗∗	0.00
NIBUT versus diabetes duration	−0.29∗	0.03
NIBUT versus TNS	−0.29∗	0.03
NIBUT versus retinopathy grade	−0.03	0.82
PRTT versus TNS	0.01	0.96
PRTT versus retinopathy grade	−0.16	0.26

∗∗Correlation is significant at the 0.01 level (2-tailed).

∗Correlation is significant at the 0.05 level (2-tailed).
